# Where Metabolism Meets Senescence: Focus on Endothelial Cells

**DOI:** 10.3389/fphys.2019.01523

**Published:** 2019-12-18

**Authors:** Jacopo Sabbatinelli, Francesco Prattichizzo, Fabiola Olivieri, Antonio Domenico Procopio, Maria Rita Rippo, Angelica Giuliani

**Affiliations:** ^1^Department of Clinical and Molecular Sciences (DISCLIMO), Università Politecnica delle Marche, Ancona, Italy; ^2^IRCCS MultiMedica, Milan, Italy; ^3^Center of Clinical Pathology and Innovative Therapy, IRCCS INRCA, Ancona, Italy

**Keywords:** cellular senescence, endothelial cells, age-related diseases, type 2 diabetes, metabolism, glycolysis

## Abstract

Despite the decline in their proliferative potential, senescent cells display a high metabolic activity. Senescent cells have been shown to acquire a more glycolytic state even in presence of high oxygen levels, in a way similar to cancer cells. The diversion of pyruvate, the final product of glycolysis, away from oxidative phosphorylation results in an altered bioenergetic state and may occur as a response to the enhanced oxidative stress caused by the accumulation of dysfunctional mitochondria. This metabolic shift leads to increased AMP/ATP and ADP/ATP ratios, to the subsequent AMPK activation, and ultimately to p53-mediated growth arrest. Mounting evidences suggest that metabolic reprogramming is critical to direct considerable amounts of energy toward specific activities related to the senescent state, including the senescence-associated secretory phenotype (SASP) and the modulation of immune responses within senescent cell tissue microenvironment. Interestingly, despite the relative abundance of oxygen in the vascular compartment, healthy endothelial cells (ECs) produce most of their ATP content from the anaerobic conversion of glucose to lactate. Their high glycolytic rate further increases during senescence. Alterations in EC metabolism have been identified in age-related diseases (ARDs) associated with a dysfunctional vasculature, including atherosclerosis, type 2 diabetes and cardiovascular diseases. In particular, higher production of reactive oxygen species deriving from a variety of enzymatic sources, including uncoupled endothelial nitric oxide synthase and the electron transport chain, causes DNA damage and activates the NAD^+^-consuming enzymes polyADP-ribose polymerase 1 (PARP1). These non-physiological mechanisms drive the impairment of the glycolytic flux and the diversion of glycolytic intermediates into many pathological pathways. Of note, accumulation of senescent ECs has been reported in the context of ARDs. Through their pro-oxidant, pro-inflammatory, vasoconstrictor, and prothrombotic activities, they negatively impact on vascular physiology, promoting both the onset and development of ARDs. Here, we review the current knowledge on the cellular senescence-related metabolic changes and their contribution to the mechanisms underlying the pathogenesis of ARDs, with a particular focus on ECs. Moreover, current and potential interventions aimed at modulating EC metabolism, in order to prevent or delay ARD onset, will be discussed.

## Introduction

Aging is the leading single risk factor for the development of most, if not all, major age-related chronic diseases, such as neurodegenerative, cancer, metabolic and cardiovascular diseases (CVDs). Aging and age-related diseases (ARDs) share a common set of basic biological mechanisms, such as inflammation, the accumulation of macromolecular damage, adaptation to molecular and psychological stressors, epigenetic changes, metabolic dysfunction, loss of proteostasis, and defective stem cell function (Kennedy et al., 2014). Major ARDs include type 2 diabetes (T2DM), cardiovascular diseases (CVDs), osteoporosis and certain types of cancers (Olivieri et al., 2018; Prattichizzo, 2019). The low-grade, chronic, and systemic inflammation underlying the aging process and ARDs was called “inflammaging” (Franceschi et al., 2000; Fulop et al., 2018).

The notion that the lifespan of many species can be extended through reduction of energy intake (Speakman, 2005) suggests a critical role of macronutrient metabolism in the control of regulatory processes influencing proliferation, survival (Redman et al., 2018; Mitchell et al., 2019), and ARD development (Fontana et al., 2004). Accordingly, obesity is a risk factor for many ARDs and carries out a life-shortening action (Poirier et al., 2006). The onset of ARDs can be counteracted through overweight reduction by decreasing the energetic food and by increasing energy expenditure with physical activity (Stubbs and Lee, 2004; Everitt and Le Couteur, 2007; Fontana and Partridge, 2015). Notably, centenarians, individuals capable of reaching the extreme limit of human life and characterized by an exceptionally healthy phenotype, share features observed in human adult volunteers who followed caloric restriction (CR) regimens (Franceschi et al., 2018). All these evidences suggest that major pathways driving organismal aging are intimately connected with metabolism (Lopez-Otin et al., 2016), a hypothesis also confirmed by the fact that dysfunctional mitochondria are a common feature of aged cells and major ARDs (Lane et al., 2015; Correia-Melo et al., 2016).

Within the cells, energy is generated in the form of adenosine triphosphate (ATP), mainly through mitochondrial oxidative phosphorylation (OXPHOS) in the presence of oxygen, and through anaerobic glycolysis in its absence (Saraste, 1999). This “general rule” could be subjected to modifications during particular conditions, such as cellular senescence (Wiley and Campisi, 2016). Senescent cells (SCs) exhibit some peculiar characteristics, including growth arrest, telomere shortening, enhanced senescence associated (SA) β-Galactosidase activity, and the acquisition of a pro-inflammatory senescence-associated secretory phenotype (SASP), which is responsible for both inflammaging and the spreading of senescence to neighboring cells. Beyond the exhaustion of replicative potential, cellular senescence can also be triggered by different stressors, including hypoxia, endotoxin, and reactive oxygen species (ROS) (Campisi, 2013).

Endothelial cells (ECs) form the inner lining of blood vessels (Fishman, 1982). In adult organisms they are supposed to remain in a quiescent state, but they can be rapidly activated by a variety of stimuli (Schlereth et al., 2018). They provide a significant contribution to the transduction of signals between blood and tissues (Munoz-Chapuli et al., 2004); moreover, by the release of the gaseous mediator nitric oxide (NO), ECs play a crucial role in maintaining the vascular tone and in preventing platelet aggregation (Huang et al., 1995; Sabbatinelli et al., 2017). The integrity of the EC monolayer is a critical requisite to allow blood flow and avoid uncontrolled thrombosis (Giannotta et al., 2013). Growing evidence show that dysfunctional senescent ECs can play a key role in instigating the morphological and biochemical changes that accompany vascular dysfunction, thus not a mere epiphenomenon in pathogenesis of CVDs (Childs et al., 2014; Tian and Li, 2014). For this reason, senescence of ECs has become a central focus of the investigations on ARDs.

Here, we review the latest findings on the metabolic changes that occur during the aging process of endothelial cells. Moreover, due to EC strategic location in the human organism, here we support the hypothesis of a potential role of senescence-associated metabolic alterations of ECs in the onset and development of ARDs. Finally, the potential relevance of targeting specific EC metabolic features to counteract ARDs will be discussed.

## Metabolic Reprogramming in Cellular Senescence

A peculiar feature of SCs is that they remain metabolically active, despite their growth arrest. Their high metabolic rate is intimately linked to SASP acquisition, but whether it is a cause or an effect of the inflammatory phenotype and altered proliferative status of SCs has yet to be established (Wiley and Campisi, 2016).

Recent literature confers to metabolic reprogramming a deterministic role in modulating the inflammatory responses of the innate immune cells (Van den Bossche et al., 2017): in macrophages, metabolic pathways not only provide energy but also regulate their phenotype and function. Lipopolysaccharide (LPS)(+IFNγ)-activated proinflammatory (i.e., M1) macrophages mediate host defense through an enhanced glycolytic metabolism and impaired mitochondrial OXPHOS. On the contrary, interleukin (IL)-4(13)-activated (i.e., M2) macrophages, which promote wound healing and Th2-mediated responses, mainly rely on OXPHOS for the synthesis of ATP. These data suggest that metabolic changes may also underlie acquisition of the SASP in senescent cells.

In the 1980s, the first attempts to analyze energy metabolism in aging cells showed that glucose consumption and lactate production are elevated in replicative senescent human diploid fibroblasts (HDFs) (Goldstein et al., 1982; Bittles and Harper, 1984). This “glycolytic state,” accompanied by an imbalance of the activity of the glycolytic enzymes, results in a less energetic state, mirrored by the drop of ATP and GTP intracellular levels as cell cultures enter in replicative senescence (Zwerschke et al., 2003). More recently, metabolomic approaches on extracellular metabolites released by senescent fibroblasts confirmed a shift toward glycolysis compared to young cells (James et al., 2015). This general mechanism, however, is more or less pronounced depending on the cell type and shows some exceptions, such as senescent human mammary epithelial cells where glucose consumption and lactate secretion do not increase (Delfarah et al., 2019).

**FIGURE 1:**
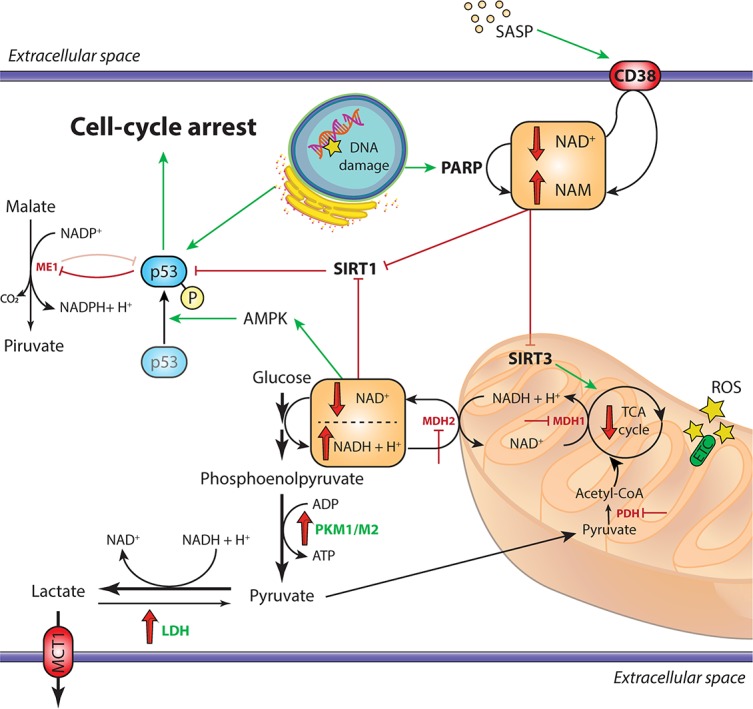
Overview of the metabolic alterations driving cellular senescence. In cells undergoing replicative senescence, the upregulation of LDH and the inhibition of both PDH and the malate-aspartate shuttle (MDH1 and MDH2) result in the diversion of pyruvate away from oxidative phosphorylation and toward aerobic glycolysis. This leads to the decrease of cytosolic NAD^+^/NADH ratio, which triggers the activation of the energy sensor AMPK. Moreover, the accumulation of DNA damage, also mediated by ROS in the dysfunctional mitochondria and the SASP, activate the NAD^+^-consuming enzymes PARP1 and CD38, respectively. The subsequent impairment of SIRT1 deacetylase activity, coupled with the AMPK-mediated phosphorylation of p53, triggers the arrest of cell replication and the establishment of irreversible senescence. Moreover, activated p53 inhibits the activity of the malic enzymes ME1 and ME2, further impairing the cellular antioxidant mechanisms through reduction of NADPH levels. Downregulated enzymes are in red, upregulated enzymes are in green; red and green arrows indicate repression or induction, respectively. AMPK, adenosine monophosphate-activated protein kinase; ETC, electron transport chain; LDH, lactate dehydrogenase; MCT1, monocarboxylate transporter 1; MDH1/MDH2, malate dehydrogenase 1/2; ME1, malic enzyme 1; PARP, poly (ADP-ribose) polymerase; PDH, pyruvate dehydrogenase; PKM1/PKM2, pyruvate kinase M1/M2; ROS, reactive oxygen species; SASP, senescence-associated secretory phenotype; SIRT, sirtuin; TCA, tricarboxylic acid.

The role of malic enzyme (ME) in senescent cells has been extensively investigated due to its role in maintaining cellular redox homeostasis. The two ME isoforms ME1 and ME2 catalyze the decarboxylation of malate to pyruvate with concomitant formation NADPH or NADH, respectively. Regeneration of reducing equivalents for anabolic processes in form of NADPH can be achieved either through the “malate oxidation shunt” or through the pentose phosphate pathway (PPP) (Fox et al., 1994). In the context of aging cell, p53 by binding to specific response elements, attenuates ME1 and ME2 transcription. At the same time ME depletion induces senescence by stabilizing p53, suggesting that also ME exerts an inhibitory effect on p53 (Jiang et al., 2013). Moreover, expression of both malate dehydrogenase (MDH)1, a mitochondrial tricarboxylic acid (TCA) cycle enzyme catalyzing malate oxidation to oxaloacetate, and MDH2, the cytosolic enzyme of malate-aspartate shuttle, declines during aging, leading to an impaired transfer of reducing equivalents into the mitochondria. In HDF, the subsequent decrease of cytosolic NAD^+^/NADH ratio is accompanied by the induction of replicative senescence (Zwerschke et al., 2003; Lee et al., 2012; Wiley et al., 2016). The need to regenerate NAD^+^ explains the upregulation of lactate dehydrogenase (LDH) in senescent HDFs, which release lactate to avoid the arrest of glycolysis due to both product accumulation and excessively low intracellular pH (Lang et al., 2003; Zwerschke et al., 2003) (Figure 1). These observations on the role of malate prompted the hypothesis that malate supplementation could delay senescence and extend lifespan (Edwards et al., 2013). Similar effects have been shown in *C. elegans* and *D. melanogaster* for α-ketoglutarate and oxaloacetate, two other TCA cycle intermediates (Williams et al., 2009; Chin et al., 2014; Su et al., 2019). However, such observation needs to be corroborated by evidence in mammals.

The role of PPP, the alternative source of NADPH, has been explored in cellular senescence. In fact, even if SCs have a lower demand of deoxyribonucleotides (dNTPs), they still require NADPH to allow the activity of the reactive oxygen species (ROS)-detoxifying thioredoxins, glutaredoxins and peroxiredoxins. Importantly, replicative senescence can be triggered by a lack of dNTPs due to reduced substrate availability. The activity of the first enzyme of the oxidative branch of PPP, namely glucose-6-phosphate dehydrogenase (G6PDH), is decreased during senescence. Accordingly, G6PDH-deficient cells exhibit accelerated oxidant-induced senescence (Ho et al., 2000; Cheng et al., 2004), a process that can be partially rescued by telomerase ectopic expression (Wu et al., 2009). Notably, transgenic mice overexpressing G6PDH display extended lifespan through increased NADPH levels (Nobrega-Pereira et al., 2016) and knockdown of the tumor suppressor ataxia-telangiectasia mutated (ATM) gene restores glucose flux throughout the PPP and allows cells to overcome senescence (Aird et al., 2015).

The findings on the metabolic features of replicative SCs have been only in part confirmed in oncogene-induced senescence (OIS). Seminal studies reported that cells undergoing OIS have high glycolysis rate, along with an elevated OXPHOS activity, when compared to proliferating cells (Quijano et al., 2012; Dorr et al., 2013; Kaplon et al., 2013; Takebayashi et al., 2015). In line with these findings, both pyruvate kinase (PK) isoforms, i.e., PKM1 and PKM2, and pyruvate dehydrogenase (PDH), two enzymes with a key role in glycolysis and in the conversion of pyruvate to acetyl-CoA, are upregulated in SCs. This leads to an enhanced use of pyruvate for the TCA cycle causing increased cellular respiration and redox stress (Zwerschke et al., 2003; Dorr et al., 2013; Kaplon et al., 2013). The TCA cycle could be also fueled by metabolites from fatty acid catabolism. Indeed, Ras-induced senescent cells manifest a decline in lipid synthesis and an increase in fatty acid oxidation (FAO), which results in a higher rate of basal oxygen consumption (Quijano et al., 2012). Inhibition of carnitine palmitoyltransferase 1A (CPT1A), the key rate-limiting enzyme for oxidation of free fatty acids (FFA) into the mitochondria, prevented senescence and SASP establishment (Quijano et al., 2012). In a recent work, Fafián-Labora et al. demonstrated that fatty acid synthase (FASN) activity is important for mitochondrial bioenergetics in the initial phases of senescence. FASN is an enzyme that catalyzes *de novo* synthesis of fatty acids by combining malonyl-CoA to the acetyl-CoA derived from glycolysis-produced pyruvate. Indeed, inhibition of FASN activity prevented the p53-mediated induction of senescence, the secretion of the canonical SASP factors IL-1α, IL-1β, IL-6, and the release of extracellular vesicles (EVs) mediating the spread of pro-senescence signals at the paracrine level (Borghesan et al., 2019; Fafian-Labora et al., 2019). Notably, other studies reported that p53 activation inhibits FASN, suggesting a negative feedback loop (Ford, 2010).

High lipogenesis would explain the progressive accumulation of membranous organelles during cell senescence (Kim et al., 2010). It is well known that lysosome mass expands during senescence, recently an increase in mitochondrial mass was also established (Correia-Melo et al., 2016; Fafian-Labora et al., 2019). A considerable number of senescent−associated changes are dependent on mitochondria. Indeed, mitochondrial dysfunction can elicit a specific type of proinflammatory phenotype, defined as mitochondrial dysfunction-associated senescence (MiDAS) (Wiley et al., 2016). MiDAS differs from the prototypical SASP for the lack of an IL-1/NF-κB-dependent mechanism. In MiDAS, a reduced NAD^+^/NADH ratio is believed to trigger adenosine monophosphate-activated protein kinase (AMPK) and p53 activation (Wiley et al., 2016; Giuliani et al., 2017).

NAD^+^/NADH ratio is one of the most reliable markers of the redox state of the cell. Its decrease has been reportedly linked to cellular senescence (Son et al., 2016; Zhang et al., 2016). NAD^+^ decline in aging is extensively reviewed by Verdin (Verdin, 2015). Low levels of NAD^+^ were also reported in several tissues (Massudi et al., 2012; Zhu et al., 2015), and supplementation of NAD^+^ precursors increased life span in different species (Fang et al., 2016; Zhang et al., 2016). Interestingly, SCs were shown to induce the SASP-mediated expression of CD38 – an ectoenzyme with a high NADase activity – in non-senescent cells, such as endothelial cells and bone marrow-derived macrophages (Chini et al., 2019; Covarrubias et al., 2019). CD38 inhibitors rescued NAD^+^ decline and ameliorated a number of age-related metabolic outcomes in mice (Tarrago et al., 2018). A similar beneficial effect on mouse lifespan has been described also for the EV-mediated cell-to-cell transfer of the extracellular isoform of nicotinamide phosphoribosyltransferase (NAMPT), the rate-limiting enzyme of NAD^+^ salvage pathway (Yoshida et al., 2019). EVs are membrane-coated nanoparticles actively released by almost all cell types, including ECs (Jansen et al., 2017). EVs are usually categorized according to their size and surface markers, and are able to shuttle and deliver functional proteins and nucleic acids in a paracrine and systemic manner (Thery et al., 2018).

Recently, Nacarelli et al. (2019) demonstrated a direct link between intracellular NAD^+^ levels and the SASP in OIS. High SA-chromatin remodeling is related to the upregulation chromatin-binding protein High-Mobility Group A1 (HMGA1) which binds genomic A + T reach regions to increase chromatin accessibility (Nacarelli et al., 2019). Interestingly, inhibition or silencing of NAMPT, one of the targets of HMGA1, decreased glycolysis, mitochondrial respiration, and oxygen consumption, along with NAD^+^/NADH ratio, which is normally elevated in OIS (Nacarelli et al., 2019; Søgaard and Gil, 2019). The subsequent increase of ADP/ATP ratio activates AMPK, which leads to p53-mediated inhibition of p38MAPK. By activating NF-κB, p38MAPK acts as an important up-stream effector of the SASP (Freund et al., 2011). Therefore, in OIS a high NAD^+^/NADH ratio finally results in the activation of the SASP. The apparently contrasting observation in different models of senescence lends support to the hypothesis that NAD^+^ metabolism specifically controls distinct subsets of SASP factors. Indeed, MiDAS is triggered by a drop of NAD^+^/NADH and leads to IL-10 and TNF-α production (Wiley et al., 2016), whereas OIS boosts NAD^+^ levels, in contrast with the general decline of NAD^+^/NADH ratio observed in aging and confirmed in replicative senescence (Zwerschke et al., 2003).

We can conclude that dramatic alterations of carbohydrate and lipid metabolism occur in senescent cells, with divergent outcomes partially depending on senescence trigger. In general, senescent cells acquire a “glycolytic state,” but the fate of the resulting pyruvate changes between replicative and oncogene/therapy induced senescence. Notably, the pyruvate “hub” has been promoted as a druggable target for treatment of many diseases, including diabetes, ischemic heart disease, and cancer (Roche and Hiromasa, 2007; Olenchock and Vander Heiden, 2013). By directing specific substrates to one pathway or another, the entire metabolic set-up of the cell can be affected, in some cases reaching a desirable effect.

## Metabolic Features of Endothelial Cells

Although they share common characteristics, ECs can exhibit several phenotypic differences, depending on the specific chemical and physical characteristics of the vascular districts in which they are living (Dejana et al., 2017). ECs in the microvasculature are involved in the bidirectional exchange of gases, macromolecules, and cells between tissues and blood, and can also perform enzymatic modifications of circulating mediators, such as lipoproteins and angiotensin I (Chi et al., 2003). ECs actively participate in angiogenesis, i.e., the generation of new capillaries from existing vessels; in the adult physiological angiogenesis occurs mainly in the female reproductive system and in wound healing, however it plays a significant role in promotion and progression of many pathological conditions, including cancer and chronic inflammation (Potente and Carmeliet, 2017). Angiogenesis implies a switch of selected ECs toward a proliferative and migratory phenotype (Jimenez-Valerio and Casanovas, 2017). These ECs are known as tip and stalk cells, the first guiding the migration of the latter to achieve the elongation of the developing vessel (Jakobsson et al., 2010). Angiogenesis is a high energy-demanding process, therefore, from their original quiescent state, ECs must undergo a substantial reprogramming of their metabolism.

Quiescent ECs rely mainly on glycolysis for their energy demanding, despite the high abundance of oxygen in the vascular compartment. Accordingly, even if the mitochondrial mass in ECs is variable depending to the vascular district; it is generally lower than other cell types (Eelen et al., 2018). During angiogenesis, vascular endothelial growth factor (VEGF) signaling induces in ECs a further boost of glycolysis, with the upregulation of the GLUT-1 transporter and an increased synthesis of the allosteric modulator fructose-2,6-bisphosphate (F2,6BP) by the enzyme 6-phosphofructo-2-kinase/fructose-2,6-bisphosphatase 3 (PFKFB3), a master regulator of glycolysis (De Bock et al., 2013). In this context, EC mitochondrial metabolism remains largely underexplored. While a substantial amount of evidence is available on the impairment of angiogenesis under glucose-deprivation conditions (He et al., 2013; Terashima et al., 2013; Garcia et al., 2015; Gu et al., 2019), only one very recent report demonstrated that also the inhibition of electron transport chain decreases EC proliferation. Interestingly, this effect is related to a lowered NAD^+^/NADH ratio, rather than to a decreased availability of ATP (Diebold et al., 2019).

The notion that ECs exhibit different glycolytic rates according to their specialization or proliferation rate is quite intuitive, however, glycolysis is not only an energy supplier for these cells but can modulate their function and phenotype. Under hypoxic conditions, the hypoxia inducible factor 1-alpha (HIF-1α)-mediated upregulation of the glycolytic pathway, coupled with the downregulation of PDH, induces an accumulation of lactate, which in turn i) stabilizes HIF-1α and mediates a paracrine proangiogenic effect on neighboring ECs (Sonveaux et al., 2012; Lee et al., 2015), ii) affects the functional polarization of tumor-associated macrophages toward the pro-tumoral M2 phenotype (Colegio et al., 2014). EC phenotype can be changed through targeting enzymes or regulators of the glycolysis. The forkhead box O transcription factor 1 (FOXO1) plays a major role in maintaining ECs in a quiescent state by suppressing c-myc signaling and reducing glycolysis (Wilhelm et al., 2016). Similarly, the downregulation of hexokinase 2 (HK2) via the disruption of the fibroblast growth factor receptor (FGFR)/c-myc axis impairs proliferation and migration of ECs (Yu et al., 2017). The interplay between the two PK isoforms represents an important crossroad in determining the fate of the resulting pyruvate. In a model of pulmonary arterial hypertension, the increased PKM2 expression fosters aerobic glycolysis and induces a more proliferative state (Caruso et al., 2017). On the contrary, under physiological conditions, the laminar blood flow promotes the NO-mediated S-nitrosylation of PKM2, which results in a reduced glycolysis and in an enhanced funneling of substrates through the PPP (Siragusa et al., 2019). In cells with a low replication rate, like quiescent ECs, a balanced PPP activity promotes antioxidant responses through synthesis of reducing equivalents in form of NADPH.

The metabolic features of senescent ECs are still poorly explored. Moreover, whether the effects of EC senescence, including the SASP, could be mediated by metabolic changes is still being debated. An emerging role for SIRT3, a member of the sirtuin family mainly localized in the mitochondria, in cellular aging has been outlined by recent studies (Ansari et al., 2017). Through its NAD-dependent deacetylase activity, SIRT3 regulates the mitochondrial metabolic pathways, including FAO (Kanwal, 2018). Specifically, SIRT3 promotes the influx of substrates into the TCA cycle by promoting the activity of PDH and acyl-CoA dehydrogenase (Kincaid and Bossy-Wetzel, 2013). Moreover, SIRT3 seems to exert a positive regulator effect on the TCA cycle, even if results are still contradictory (Verdin et al., 2010). Similarly to the other members of the sirtuin family, depletion of SIRT3 has been shown to reduce human lifespan (Bellizzi et al., 2005; Brown et al., 2013). Notably, strategies aimed to upregulate SIRT3 in endothelial cells resulted in a higher protection against stress-induced premature senescence, an effect mediated by the deacetylation of FoxO3 (Liu et al., 2015; Xing et al., 2018). For some aspects, ECs seem to escape the rules describing the senescence-associated metabolic shift that have been postulated from studies in other cell lineages. For example, the observation that HDF senescence is accompanied by an upregulation of the whole glycolytic machinery was not confirmed in HUVECs (Unterluggauer et al., 2008). Conversely, a recent report showed a senescence-associated decline in EC glycolysis, which is mediated by a reduced PFKFB3 activity. This trend, but not senescence, was reverted by nuclear factor erythroid 2-related factor 2 (NRF2) overexpression (Kuosmanen et al., 2018). Another study on EC replicative senescence revealed that a NAMPT/SIRT1/FoxO1-mediated slight increase in aerobic glycolysis exerts a protective effect by limiting ROS production (Borradaile and Pickering, 2009).

Glutamine represents another important metabolite for ECs. It can be used as an energy source, via deamination and subsequent transamination to form α-ketoglutarate which enters the TCA cycle, to produce the antioxidant peptide glutathione, or to provide substrates for nucleotide biosynthesis (Eelen et al., 2015). Glutamine was shown to be required for vessel sprouting, even if the impaired angiogenesis during glutamine starvation could be rescued by asparagine supplementation (Huang H. et al., 2017). The notion that senescent ECs strongly rely on glutaminolysis for their energy demand comes from the observation in these cells of an increased lactate synthesis independent from glycolysis. Remarkably, the inhibition of glutaminase 1 (GLS1) is able to induce apoptosis and senescence even in young ECs (Unterluggauer et al., 2008).

Although ECs obtain a minor amount of ATP from oxidative phosphorylation, they efficiently oxidize fatty acids: ECs are endowed with all the proteins required for the uptake and intracellular transport of fatty acids (Hagberg et al., 2013). Indeed, in the microcirculation, ECs can extract fatty acids from circulating lipoproteins through lipoprotein lipase (LPL). Fatty acids are then absorbed through the fatty acid transport proteins (FATP)3 and FATP4 and bound by the intracellular fatty acid binding protein FABP4. Fatty acids can then be oxidized to provide carbons to replenish TCA cycle, thus allowing the synthesis of dNTP precursors, or directed to the surrounding tissues (Mehrotra et al., 2014). In a recent report, Kalucka et al. showed that fatty acid oxidation is the only upregulated metabolic pathway in quiescent ECs. Notably, beta-oxidation is increased neither for bioenergetic purposes nor to meet the anabolic demands of the cells. Rather, fatty acids are oxidized to increase NADPH regeneration by the malic enzyme once their carbons enter the TCA cycle in form of acetyl-CoA. As a result, quiescent ECs have higher amounts of reduced glutathione and thus are more protected against oxidative stress (Kalucka et al., 2018). Interestingly, this beneficial effect is mediated by Notch1 signaling, which exerts also a central role during the earlier phases of senescence, by switching the secretome of ECs away from the pro-inflammatory SASP and toward the TGF-β-mediated release of immunosuppressive and fibrogenic factors (Hoare et al., 2016).

Cellular senescence also involves endothelial progenitor cells (EPCs), a population of circulating CD34^+^ cells participating in new vessel formation and in vascular remodeling through their ability to differentiate into ECs. The age-related decline in EPC survival and regeneration could precipitate endothelial dysfunction, thus triggering the onset of CVDs (Olivieri et al., 2016). It has been demonstrated that the increased susceptibility of elderly individuals to ischemic disorders could be mediated, at least in part, by a blunted response of senescent EPCs to hypoxia, which fail to upregulate selected genes related to HIF-1 signaling and glucose uptake (Wu et al., 2018). To this regard, the upregulation of SIRT1 and NRF2 have both been shown to delay EPC senescence and to preserve the proliferative, migratory, and angiogenic activities in senescent EPCs (Lamichane et al., 2019; Wang et al., 2019).

Investigations on EC metabolic features could take advantage of their peculiar localization and lend important insight into the pathogenesis of CVDs. Targeted metabolomics of large cohorts of samples are required to get a more comprehensive point of view on mechanisms which were extensively characterized in *in vitro* models. While multiomics approaches proved useful to identify circulating signatures with a diagnostic and/or prognostic role for many conditions (Zierer et al., 2015; Correia et al., 2017; Niewczas et al., 2019), their role in providing *ex vivo* mechanistic clues is hampered by the lack of information on the relative contribution of various tissues to the circulating metabolome. To address this issue, the field is moving toward more specific approaches allowing the deconvolution of complex circulating signatures, including the application of machine learning tools and the study of EVs (Heitzer et al., 2019). A growing body of evidence is focusing on the characterization of endothelial EVs isolated from plasma (Igami et al., 2019; Mork et al., 2019; Oliveira et al., 2019; Utermohlen et al., 2019). Even if a consensus on the methods for isolation and characterization of these EVs is far from being reached, it is important to remark that the profiling of the cargo of circulating EVs sorted according to the parent cells allows enhanced specificity compared to single molecular markers in the blood.

**FIGURE 2:**
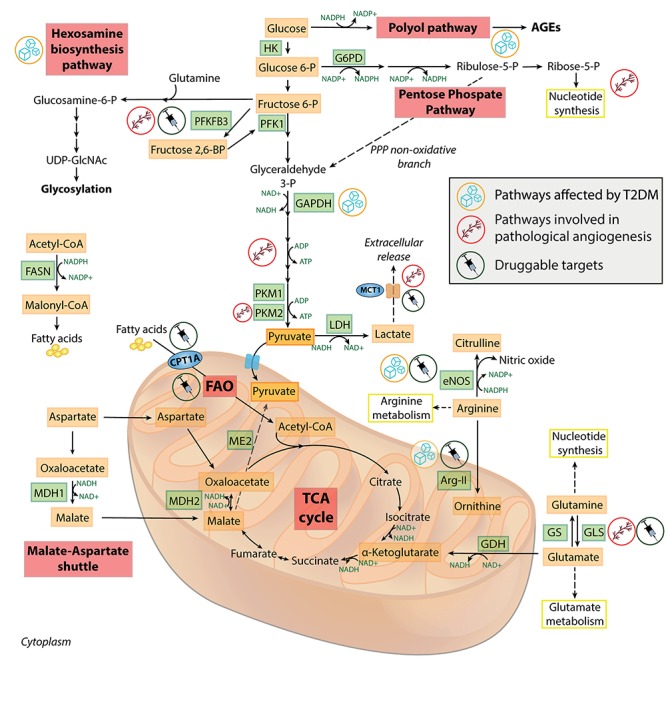
Metabolic features of healthy and dysfunctional endothelial cells. Schematic overview of the most relevant metabolic pathways in endothelial cells. Pathways, enzymes and metabolites affected by T2DM or involved in pathological angiogenesis, and the druggable targets discussed in the text, are labeled by specific icons. AGEs, advanced glycation end-products; Arg-II, arginase II; CPT1A, carnitine palmitoyltransferase 1A; eNOS, endothelial nitric oxide synthase; FAO, fatty acid oxidation; FASN, fatty acid synthase; G6PD, glucose 6-phosphate dehydrogenase; GAPDH, glyceraldehyde 3-phosphate dehydrogenase; GDH, glutamate dehydrogenase; GLS, glutaminase; GS, glutamine synthetase; HK, hexokinase; LDH, lactate dehydrogenase; MCT1, monocarboxylate transporter 1; MDH1/MDH2, malate dehydrogenase 1/2; ME2, malic enzyme 2; PFK1, phosphofructokinase 1; PFKFB3, 6-phosphofructo-2-kinase/fructose-2,6-biphosphatase 3; PKM1/PKM2, pyruvate kinase 1/2; PPP, pentose phosphate pathway; T2DM, type 2 diabetes; TCA, tricarboxylic acid; UDP-GlcNAc, uridine diphosphate N-acetylglucosamine.

Figure 2 provides an overview of the most relevant physiologic and pathologic pathways in ECs. Understanding the mutual influence between EC senescence and the complex network of metabolic pathways could provide valuable insight into the pathogenesis of many ARDs. This will be the focus of the next sections.

## Endothelial Cell Metabolism in Age-Related Diseases

Increasing evidence is suggesting a bidirectional interplay among EC metabolism and ARDs (Graupera and Claret, 2018). One of the most characterized metabolic alteration in ECs exposed to hyperglycemia, i.e., a known senescence-promoting stimulus (Prattichizzo et al., 2018a), is the diversion of glycolytic intermediates into alternate pathways (Figure 2). This is the result of the activation of the enzyme polyADP-ribose polymerase 1 (PARP1) following the ROS-induced DNA damage. PARP1 mediates the ribosylation of glyceraldehyde 3-phosphate dehydrogenase (GAPDH) and depletes NAD^+^ intracellular levels, further inhibiting the glycolysis flux (Du et al., 2003). Moreover, entry of glucose 6-phosphate into the PPP is also restricted, due to a cAMP-mediated impairment of glucose 6-phosphate dehydrogenase during hyperglycemia (Zhang et al., 2010). The reduced PPP activity leads to lowered NADPH levels, causing both a reduced endothelial nitric oxide synthase (eNOS) activity and a blunted antioxidant response. In addition, excessive glucose is metabolized into the pathologic polyol and hexosamine biosynthetic pathways, which are the major contributors to the generation of advanced glycation end-products in diabetes (Berrone et al., 2006). The aberrant non-enzymatic glycation of circulating and intracellular proteins is a key determinant of T2DM cardiovascular complications (Mapanga and Essop, 2016). Endothelial dysfunction (ED) and senescence are “historically” considered two consequences of T2DM (Bonfigli et al., 2016; Prattichizzo et al., 2018a, d), possibly as a result of the overabundance of senescence-promoting, endothelium-damaging factors in the bloodstream of these patients, e.g., pro-oxidant molecules, glucose, and lipids (Prattichizzo et al., 2018c). In particular, the role of EC senescence has been emphasized in both the development of T2DM and of its CV complications. Indeed, EC senescence has been found in the adipose tissue of obese subjects preceding the development of T2DM (Minamino et al., 2009; Villaret et al., 2010), as well as in other vascular districts in diabetic mice and humans (Orimo et al., 2009; Prattichizzo et al., 2018a; Yokoyama et al., 2019). On the other side, the observation that ED development often precedes the appearance of the T2DM phenotype in obese subjects (Hadi and Suwaidi, 2007), coupled by the intuitive assumption that the endothelium represents the biggest organ regulating nutrient availability across the whole organism, has prompted the research toward the study of the effect of selective gain/loss of function of metabolic genes in ECs (Graupera and Claret, 2018).

A seminal paper suggested that deletion of insulin receptor (IR) in ECs is sufficient to alter the expression of eNOS and endothelin-1 in mice treated with a high-salt diet, providing the first link among EC metabolism and general vascular tone (Vicent et al., 2003). Later, another group showed that EC-specific Irs2 KO mice have a blunted insulin-dependent glucose uptake in the muscles when fed with HFD (Kubota et al., 2011). Beyond insulin signaling, also the selective modulation in ECs of genes involved in angiogenesis, i.e., VEGF/VEGFR2, ANG2/TIE2, and DLL4/NOTCH1 (Robciuc et al., 2016; Seki et al., 2018), as well as in fatty acids transport, i.e., VEGF-B/NRP1 and CD36, affect whole body metabolism (Hagberg et al., 2010; Son et al., 2018). Interestingly, both promotion and inhibition of angiogenesis have been shown to foster insulin sensitivity, suggesting a context-dependent effect (Sun et al., 2012). Proposed mechanistic explanations include the induction of apoptosis in dysfunctional adipocyte (for restricted angiogenesis) and “healthy” expansion of adipose tissue (for enhanced angiogenesis) (Graupera and Claret, 2018).

While the role of senescent EC metabolism has not been studied in relation to specific ARDs, a key role for an alteration of metabolism in senescent ECs may be inferred by studies modulating key factors involved in the senescence process. Indeed, mice with EC-specific p53 deficiency fed a high fat diet (HFD) showed improvement of insulin sensitivity and less fat accumulation compared to WT mice (Yokoyama et al., 2014). Of note, p53 is a master transcription factor promoting senescence in virtually all cell types (Hafner et al., 2019). Similarly, NF-κB activation is held to be a cornerstone of SASP development in a plethora of different SCs, including ECs (Salminen et al., 2012). Transgenic mice expressing dominant-negative IκB under the Tie2 promoter/enhancer (thus with functional inhibition of NF-κB signaling specifically in ECs) were protected from the development of insulin resistance associated with both genetic and diet-induced obesity. Strikingly, these mice showed also an increase in lifespan, coupled by a decreased age-related insulin resistance and vascular senescence (Hasegawa et al., 2012). The relevance of these findings for human ARDs remains to be tested. However, an increased expression of p53, along with an increased activation of NF-κB has been observed in aged arteries from human subjects (Donato et al., 2008; Morgan et al., 2013).

Beyond T2DM and vascular function, also heart remodeling has been shown to be affected by metabolic alterations in ECs. Indeed, genetic ablation of Rbp-jκ (Notch signaling) in ECs promoted alterations in fatty acid metabolism in the whole organism, followed by heart hypertrophy and failure (Jabs et al., 2018). The deprivation of available FFAs as substrate prompted the use of glucose in cardiomyocytes and the consequent activation of the mTOR pathway, a master metabolic rheostat regulating both cellular senescence and organismal aging (Weichhart, 2018). Accordingly, a ketogenic diet was able to restore normal cardiac function in this mouse model (Jabs et al., 2018), suggesting the potential of dietary intervention to treat (and not only prevent) also life-threating ARDs. A similar framework has been proposed also to explain the cardioprotective properties of sodium-glucose cotransporter (SGLT)-2 inhibitors (i) (Ferrannini et al., 2016). SGLT-2i are a recently introduced class of glucose-lowering drugs inhibiting the reabsorption of glucose in the proximal convoluted tubule, thus promoting glucose elimination through the kidneys (Santos-Gallego et al., 2019). Clinical trials have shown a striking benefit in terms of CV mortality and worsening of heart failure in diabetic patients treated with SGLT-2i, an effect not ascribable to an improved glycemic control (Prattichizzo et al., 2018b). It has been hypothesized that the decreased availability of glucose, coupled by an increase in the circulating levels of ketone bodies (KB), improves the energetic function of the heart (Ferrannini et al., 2016; Prattichizzo et al., 2018b). This hypothesis has been recently tested in porcine, non-diabetic hearts, where the treatment with a SGLT-2i switches myocardial fuel utilization away from glucose toward KB, FFA, and branched chain amino acids, thereby improving myocardial energetics, enhancing left ventricular systolic function, and ameliorating adverse left ventricle remodeling (Santos-Gallego et al., 2019).

The promotion of myocardial function by shifting toward FFA utilization is likely dependent on the function of the liver, rather than EC metabolism. However, EC metabolism may influence the pathobiology of different organs by at least 3 mechanisms: (i) by releasing active secondary mediators, such as NO (Kubota et al., 2011; Yokoyama et al., 2014); (ii) by regulating the flow of lipids from bloodstream to organs (Hagberg et al., 2010; Jabs et al., 2018); (iii) by regulating vessel density, and thus indirectly nutrients availability and interstitial insulin levels (Robciuc et al., 2016). Beyond adipose tissue and muscle insulin sensitivity, the latter mechanism has been shown to be crucial for the development of age-induced osteoporosis (Ramasamy et al., 2016). Indeed, skeletal blood flow and endothelial Notch activity are reduced in aged mice, leading to decreased angiogenesis and osteogenesis, which can be reverted by genetic reactivation of Notch (Ramasamy et al., 2016). As mentioned above, Notch is regarded as a central metabolic sensor and regulator in multiple cell types.

Regarding possible secondary mediators released by ECs, the role of EVs deserves particular attention. The effects of EVs are now attracting intense interest also in the context of aging and ARDs (Prattichizzo et al., 2019). For instance, senescent ECs secrete miR-31 enriched EVs to inhibit mesenchymal stem cells osteogenic differentiation, possibly contributing to age-induced osteoporosis (Weilner et al., 2016). Of note, miR-31 is upregulated by high-glucose and inhibits cell differentiation also in other contexts (Zhen et al., 2017).

Overall, increasing evidence is suggesting a key role for EC metabolism in a plethora of ARDs, while less studies are available regarding the effect of the specific alterations of senescent EC metabolism in the whole organisms. However, the emerging roles of endothelial p53 and NF-κB, two cornerstones of the senescence process, in the regulation of both cellular and organismal metabolism suggest that metabolic alterations in specific senescent cells, and in particular ECs, can affect whole body metabolism and ARD development, a hypothesis deserving exploration in the future.

## Therapeutic Interventions Targeting Endothelial Cell Metabolism

Thirty years ago, the identification and cloning of VEGF paved the way to the development of novel strategies aimed to treat those conditions in which angiogenesis plays a dominant role (Apte et al., 2019). Agents targeting members of the VEGF family and their receptors are currently routinely employed in the treatment of many solid malignancies, including colorectal cancer, renal cell carcinoma, and non-small cell lung carcinoma (Tirumani et al., 2015), and in non-neoplastic conditions with a recognized angiogenic component, such as proliferative diabetic retinopathy and age-related macular degeneration (Virgili et al., 2018). Investigations on the mechanism of action of anti-VEGF agents helped to highlight the aforementioned interesting connections between angiogenesis and EC metabolism and offered a number of novel druggable targets that could prove useful, for example, in the case of failure of anti-VEGF treatments.

Targeting the high-glycolytic state of ECs within tumor vessels is emerging as an anti-cancer therapeutic strategy (Fitzgerald et al., 2018). In a murine model, pharmacological inhibition of glycolysis activator PFKFB3 promotes the normalization of tumor vessels and facilitates delivery of chemotherapeutic drugs. Moreover, PFKFB3 tightened ECs by upregulating vascular endothelium (VE)-cadherin, thus reducing the passage of cancer cells across the EC monolayer (Cantelmo et al., 2016). Interestingly, inhibition of PFKFB3 lowered the expression of adhesion molecules in ECs treated with IL-1β, suggesting that limiting glycolysis could represent a feasible approach to prevent the SASP-mediated spreading of EC senescence. PFKFB3 inhibitors recently reached the clinical trial phase, with promising results from a phase 1 multi-center study conducted on patients with solid tumors (Redman et al., 2015).

Given the proangiogenic role of lactate in ECs, several studies focused on target the monocarboxylate transporter 1 (MCT1) to avoid lactate exchange across ECs. Lactate mediates angiogenesis through the activation of the NF-κB/IL-8 pathway and the stabilization of HIF-1α (Vegran et al., 2011; Sonveaux et al., 2012). Evidence on cell and animal models revealed that MCT1 inhibition in ECs can drive direct anti-angiogenic effects through the enhanced degradation of HIF-1α. Drugs targeting MCT1 in a non-selective manner are currently been tested in small-scale trials (Kershaw et al., 2015). On the other hand, administration of the telomerase activator TA-65 contributes to improve blood flow recovery through increasing expression of HIF-1α, VEGF-A, and peroxisome proliferator-activated receptor (PPAR)-γ coactivator 1-alpha (PGC-1α), indicating that telomerase activation could prove a valuable therapeutic option to rescue ischemic tissues in elderly individuals (Kokubun et al., 2019).

Targeting EC metabolism could prove useful in treating a plethora of conditions sharing endothelial dysfunction as a pathogenic mechanism. Evidence of an age-related impairment of endothelial function dates back to 1990s. The identification of eNOS as the enzyme responsible for NO synthesis prompted the supplementation of its precursor L-arginine to reverse endothelial dysfunction (Chauhan et al., 1996). However, the enthusiasm for this essential amino acid as an easy therapeutic option in the prevention of many CVDs was tempered by the observation that L-arginine could even exert detrimental effects on vascular function through an inductive effect on arginase, which competes with NOSs for their common substrate (Hayashi et al., 2006; Schulman et al., 2006; Caldwell et al., 2015). For this reason, attention has moved to arginase as a putative target to ameliorate age-related endothelial dysfunction. A major contribution of its activity in determining microvascular dysfunction and remodeling has been outlined in obesity (Chung et al., 2014; Masi et al., 2018), arterial hypertension (Michell et al., 2011), and T2DM (Shemyakin et al., 2012). Interestingly, knockout of the gene encoding for the mitochondrial arginase type II (Arg-II) has been shown to restore eNOS function, to counteract the SASP in senescent ECs (Wu et al., 2015), and to extend lifespan in mice through the inhibition of mTOR signaling (Xiong et al., 2017). Moreover, mTOR blockade with rapamycin decreases the expression of the arterial senescence marker p19 and ameliorates oxidative stress- mediated endothelial dysfunction in old mice, suggesting a possible role for the rapamycin analogs, i.e., rapalogs, in the treatment of age-related CVDs (Lesniewski et al., 2017).

Fenofibrate, a PPAR-α agonist, is a common lipid-lowering drug exerting a number of interesting pleiotropic effects. By increasing EC eNOS expression and lowering circulating oxidized LDL, fenofibrate ameliorated the age-related endothelial dysfunction in a cohort of healthy individuals (Walker et al., 2012). Further mechanistic studies on animal models revealed that the eNOS stimulation, along with other fenofibrate effects, is mediated by enhanced AMPK activity following liver kinase B1 (LKB1) translocation from the nucleus to the cytoplasm (Sohn et al., 2017; Xin et al., 2019; Xu et al., 2019). Additionally, fenofibrate lowered the cyclooxygenase 2-mediated production of vasoconstrictor prostaglandins (Xu et al., 2019). By restoring the balance between vascular relaxation and contractility, fenofibrate could represent a feasible preventive approach for the vascular complications of T2DM. Notably, fenofibrate ameliorated osteoarthritis in elderly patients by selectively clearing senescent chondrocytes (Nogueira-Recalde et al., 2019). The observation of a similar senolytic effect on ECs would provide additional benefits for this largely employed drug also in the treatment of a number of age-related vascular conditions.

Investigations into a variety of diseases highlighted a role for CPT1, the rate-limiting enzyme for FAO, as a druggable target (Dai et al., 2018; Melone et al., 2018). Inhibition of endothelial CPT1a impairs EC proliferation and activates the endothelial-to-mesenchymal transition, which plays a role in the pathogenesis of pulmonary arterial hypertension, atherosclerosis, and tumor spreading (Schoors et al., 2015; Xiong et al., 2018). On the contrary, stimulation of CPT1 activity by chronic L-carnitine administration improved endothelial function in an animal model of congenital heart defect (Sharma et al., 2013). In light of the crucial role of FAO in maintaining EC quiescence and redox balance, L-carnitine supplementation, by counteracting the age-related decline in CPT1a activity (Gomez et al., 2012), could prove useful to delay the onset of age-related endothelial dysfunction. The encouraging evidence from several *in vitro* and animal studies still needs to be supported by large clinical trials (Bueno et al., 2005; Miguel-Carrasco et al., 2010; Ning and Zhao, 2013).

Glutamine, the most abundant circulating amino acid, has been extensively studied in aging research. Its blood levels decline during acute illness are increased in healthy centenarians (Montoliu et al., 2014; Meynial-Denis, 2016). A seminal study showed that glutamine administration in rats improves eNOS activity and reduces the endothelial inflammatory response following cardiopulmonary bypass (Hayashi et al., 2002). The activity of glutamine synthetase is impaired during endothelial dysfunction, due to peroxynitrite-mediated nitration of its active site (Gorg et al., 2005). This could lead to reduced TCA cycle anaplerosis, which results in the impairment of EC antioxidant system (see above) (Addabbo et al., 2013). Of note, restoring the glutamine-dependent anaplerosis through GLS overexpression has already proved beneficial in ameliorating the age-related bone loss (Huang T. et al., 2017), and in delaying EC senescence (Unterluggauer et al., 2008). Finally, the evidence that glutamine metabolism is also involved in EC proliferation supports the hypotheses that targeting GLS1 could represent a feasible approach to treat diseases associated with an aberrant EC proliferation (Peyton et al., 2018), and that glutamine supplementation can foster endothelial progenitor cell mobilization and promote vascular endothelium repair in diabetes-related ischemic injury (Su et al., 2017).

The observations discussed in the present section are summarized in Table 1. Altogether, these evidences reinforce the notion that alterations in EC metabolism are rather primary drivers than consequences of disease (Goveia et al., 2014). The development of drugs capable of targeting specific enzymes or pathways at the endothelial level is still in its infant phase. However, their progression into large clinical trials is imminent and could represent a turning point in the treatment of CVDs.

**TABLE 1:** Summary of the interventions targeting endothelial cell metabolism with a potential role in the treatment of age-related diseases.

**Pathway/mechanism**	**Intervention**	**Experimental model**	**Outcome(s)**	**References**	**Progression to the clinical trial stage**
Glycolysis	Genetic inhibition of PFKFB3	Tumor ECs from C57BL/6 mice livers	Tightening of the vascular barrier, decreased expression of cancer cell adhesion molecules in ECs, improved delivery of chemotherapeutic drugs	Cantelmo et al., 2016	Phase 1 NCT02044861
Hypoxia response	MCT1 inhibition	HUVECs, RJ:NMRI mice	Inhibition of HIF-1-dependent angiogenesis	Sonveaux et al., 2012	Phase 1 NCT01791595
	Administration of telomerase activator TA-65	C57BL/6 mice	Enhancement of collateral vascular flow recovery during age-related ischemia	Kokubun et al., 2019	Phase 1 NCT02766790, NCT02531334, NCT01753674, NCT02731807
Aminoacid metabolism	Arginase II knockout	HUVECs, C57BL/6J mice	eNOS recoupling, inhibition of EC SASP	Wu et al., 2015	Phase 1 NCT02009527, NCT02903914, NCT03314935, NCT03361228
		C57BL/6J mice	Extended lifespan *via* inhibition of p16^INK4a^, p66^Shc^, and S6K1 signaling pathways	Xiong et al., 2017	
	Glutamine administration	Sprague-Dawley rats	Attenuation of cardiopulmonary bypass-induced inflammatory response *via* regulation of NOSs activity	Hayashi et al., 2002	Commercially available as food supplement
		STZ diabetic C57BL/6 mice	Enhancement of circulating EPC mobilization *via* increase of plasma MMP-9, SDF-1, HIF-1 and VEGF levels	Su et al., 2017	
	Glutaminase overexpression	HUVECs	Delaying of EC senescence	Unterluggauer et al., 2008	No
	Glutaminase-1 inhibition	HUVECs, HAECs, HMECs	Inhibition of aberrant EC proliferation and migration	Peyton et al., 2018	Phase 1 and 2 (18 trials)
Fatty acid metabolism	Fenofibrate administration	Middle-aged/older men and women	Improvement of endothelium-dependent vasodilation, reduction of plasma oxLDL	Walker et al., 2012	Commercially available for the treatment of dyslipidemia
		HFD C57BL/6J mice	Inhibition of HFD-induced insulin resistance and kidney injury *via* AMPK activation	Sohn et al., 2017	
		MAECs, STZ diabetic C57BL/6 mice	Decreased intracellular O_2_^–^ levels, improvement of endothelium-dependent relaxation *via* enhanced eNOS and AMPK phosphorylation	Xin et al., 2019	
		STZ diabetic C57BL/6 mice	Amelioration of vascular endothelial dysfunction, reversal of kidney injury	Xu et al., 2019	
	Genetic and pharmacological inhibition of CPT1A	HUVECs, C57BL/6 mice	Inhibition of pathological ocular angiogenesis	Schoors et al., 2015	No
	L-carnitine administration	Hypertensive Wistar Kyoto rats	Improvement of endothelial function *via* enhanced NO and PGI_2_ bioavailability and upregulation of the antioxidant systems	Bueno et al., 2005; Miguel-Carrasco et al., 2010	Commercially available as food supplement
		HAECs	Stimulation of eNOS activity *via* AMPK/Src-mediated signaling	Ning and Zhao, 2013	
mTOR pathway	Rapamycin administration	B6D2F1 mice	Improvement of age-related endothelium-dependent vasodilation, amelioration of arterial senescence markers	Lesniewski et al., 2017	Commercially available as immunosuppressive drug

*HAECs, human aortic endothelial cells; HMECs, human dermal microvascular endothelial cells; MAECs, mouse aortic endothelial cells; HUVECs, human umbilical vein endothelial cells; PGI_2_, prostaglandin I_2_; STZ, streptozocin; S6K1, ribosomal protein S6 kinase beta-1.*

## Conclusion and Future Perspectives

Accumulating evidence is suggesting that SCs are characterized by a deep reshaping of metabolic pathways. The overall picture appears highly complex, considering that specific metabolic alterations characterize different cell types and various pro-senescence stimuli. However, a general trend toward a more glycolytic state in senescent cells has been reproduced with different stimuli and in a wide range of cell types, including ECs. The role of EC metabolism in the development of T2DM and CVDs is clearly emerging, mainly thank to specific mouse models with tissue-selective deletion of metabolic genes (Graupera and Claret, 2018). However, less information is available regarding the role of senescent EC metabolism in ARDs development, even though preliminary findings suggest a key role for senescence EC related genes, e.g., p53 and NF-κB, in the regulation of organismal aging (Hasegawa et al., 2012; Yokoyama et al., 2014). Disentangling the effective contribution of altered EC metabolism in humans remains challenging, as well as the impact of dietary or pharmacological intervention on this very specific process. Studies focusing on senescent EC metabolism are warranted to clarify the disease-modifying/preventive potential of both these approaches. In fact, interventions aimed at modifying diet and metabolism have already proven to be potentially effective strategies in the prevention and treatment of ARDs (Fontana and Partridge, 2015; Brandhorst and Longo, 2019). In particular, the preventive and curative roles of specific diets have now been demonstrated by placebo-controlled, randomized clinical trials showing a cardioprotective role for Mediterranean diet (Estruch et al., 2018) and the reduction of multiple cardiometabolic risk factors with CR (Kraus et al., 2019). While future studies will define to what extent these effects can be obtained with specific molecules/nutrients, the quality of evidence regarding these dietary patterns and ARD prevention should prompt the adoption of a Mediterranean-based, low-calories dietetic regimen at the population level.

## Author Contributions

JS, FP, and AG collected relevant literature and wrote the manuscript. JS and AG prepared the figures. FO, AP, and MR made a substantial, direct, and intellectual contribution through their experience in the field. MR critically advised and reviewed the manuscript.

## Conflict of Interest

The authors declare that the research was conducted in the absence of any commercial or financial relationships that could be construed as a potential conflict of interest.
